# P-1772. Reduction in Vancomycin-Associated Acute Kidney Injury with Montelukast

**DOI:** 10.1093/ofid/ofae631.1935

**Published:** 2025-01-29

**Authors:** Nicholas Teran, Kady Phe, Vincent Tam

**Affiliations:** Baylor St. Luke's Medical Center/University of Houston College of Pharmacy, Houston, Texas; Baylor St. Luke's Medical Center, Houston, Texas; University of Houston College of Pharmacy, Houston, Texas

## Abstract

**Background:**

Vancomycin is the most common antimicrobial used for the treatment of serious infections implicated by β-lactam resistant Gram-positive bacteria but has been associated with acute kidney injury (AKI) in up to 43% of cases. Reduction in renal injury associated with nephrotoxins has been reported in an animal model with adjuvant montelukast. However, it is unclear if this protective effect could be translated to improve patient outcomes. The objective of this study was to ascertain if montelukast is correlated with reduced vancomycin-associated AKI.

**Methods:**

We conducted a retrospective study of adults (≥ 18 years) who received intravenous vancomycin at our institution between 01/2020-01/2024. Patients were included if they met these criteria: vancomycin therapy ≥ 3 days and baseline serum creatinine (SCr) < 1.5 mg/dL. Patients who received concomitant montelukast (intervention) were compared to those without (control). The primary outcome was the prevalence of AKI (rise in SCr ≥ 1.5x baseline) as defined by Risk, Injury, Failure, Loss, or End-stage kidney disease (RIFLE) criteria. The primary outcome was compared using the Fisher’s exact test while the duration of vancomycin therapy was compared by the Student’s t test. Independent risk factors associated with AKI were identified by multivariate logistic regression.

**Results:**

Of 1739 patients screened, 110 patients were included in the intervention arm and 330 in the control. Patients received vancomycin for similar durations (mean ± SD, 5.1 ± 4.1 vs 5.9 ± 3.3 days, respectively; *P* = 0.06) and serum trough concentrations were comparable (*P* = 0.39). The intervention cohort experienced 2.7% AKI compared with 10.6% in the control (*P* = 0.01). The multivariate logistic regression analysis revealed that weight (OR, 1.02; 95% CI, 1.004 to 1.03; *P* = 0.011) and intensive care unit admission (OR, 6.9; 95% CI, 2.8 to 17.1; *P* < 0.001) were independently associated with acute kidney injury, while montelukast was protective (OR, 0.29; 95% CI, 0.09 to 0.98; *P* = 0.046).

**Conclusion:**

This study suggests that montelukast may be protective against vancomycin-associated nephrotoxicity, which may reduce patient harm and healthcare costs. Additional studies to investigate the nephroprotective mechanism will be conducted to corroborate these findings.

**Disclosures:**

**All Authors**: No reported disclosures

